# Effect of prone positioning on end-expiratory lung volume, strain and oxygenation change over time in COVID-19 acute respiratory distress syndrome: A prospective physiological study

**DOI:** 10.3389/fmed.2022.1056766

**Published:** 2022-12-02

**Authors:** Olcay Dilken, Emanuele Rezoagli, Güleren Yartaş Dumanlı, Seval Ürkmez, Oktay Demirkıran, Yalım Dikmen

**Affiliations:** ^1^Department of Intensive Care, Cerrahpaşa Faculty of Medicine, Istanbul University-Cerrahpaşa, Istanbul, Turkey; ^2^School of Medicine and Surgery, University of Milano-Bicocca, Monza, Italy; ^3^Department of Emergency and Intensive Care, ECMO Center, ASST Monza, San Gerardo University Teaching Hospital, Monza, Italy

**Keywords:** acute respiratory distress syndrome, COVID-19, prone position, end-expiratory lung volume, oxygenation, nitrogen washin/washout

## Abstract

**Background:**

Prone position (PP) is a recommended intervention in severe classical acute respiratory distress syndrome (ARDS). Changes in lung resting volume, respiratory mechanics and gas exchange during a 16-h cycle of PP in COVID-19 ARDS has not been yet elucidated.

**Methods:**

Patients with severe COVID-19 ARDS were enrolled between May and September 2021 in a prospective cohort study in a University Teaching Hospital. Lung resting volume was quantitatively assessed by multiple breath nitrogen wash-in/wash-out technique to measure the end-expiratory lung volume (EELV). Timepoints included the following: Baseline, Supine Position (S1); start of PP (P0), and every 4-h (P4; P8; P12) until the end of PP (P16); and Supine Position (S2). Respiratory mechanics and gas exchange were assessed at each timepoint.

**Measurements and main results:**

40 mechanically ventilated patients were included. EELV/predicted body weight (PBW) increased significantly over time. The highest increase was observed at P4. The highest absolute EELV/PBW values were observed at the end of the PP (P16 vs S1; median 33.5 ml/kg [InterQuartileRange, 28.2–38.7] vs 23.4 ml/kg [18.5–26.4], *p* < 0.001). Strain decreased immediately after PP and remained stable between P4 and P16. PaO_2_/FiO_2_ increased during PP reaching the highest level at P12 (P12 vs S1; 163 [138–217] vs 81 [65–97], *p* < 0.001). EELV/PBW, strain and PaO_2_/FiO_2_ decreased at S2 although EELV/PBW and PaO_2_/FiO_2_ were still significantly higher as compared to S1. Both absolute values over time and changes of strain and PaO_2_/FiO_2_ at P16 and S2 versus S1 were strongly associated with EELV/PBW levels.

**Conclusion:**

In severe COVID-19 ARDS, EELV steadily increased over a 16-h cycle of PP peaking at P16. Strain gradually decreased, and oxygenation improved over time. Changes in strain and oxygenation at the end of PP and back to SP were strongly associated with changes in EELV/PBW. Whether the change in EELV and oxygenation during PP may play a role on outcomes in COVID-ARDS deserves further investigation.

**Clinical trial registration:**

[www.ClinicalTrials.gov], identifier [NCT 04818164].

## Introduction

Lung protective ventilation with low tidal volumes and driving pressures (1), higher levels of positive end-expiratory pressure (PEEP) (2, 3) and prone positioning (PP) (4) are recommended treatments in classical ARDS (5, 6).

Prone positioning improves ventilation-perfusion matching and oxygenation in classical ARDS (7–9). A single PP session for at least 16 h (10, 11) or longer (12) is recommended to achieve a clinical benefit (13). These recommendations on the duration of PP sessions were mainly hinged on the PROSEVA trial (4) and a subsequent meta-analysis (14) that showed an improved survival in patients with ARDS.

Recently, Protti et al. reported that PP improved alveolar collapse, decreased hyperinflation and homogenized lung aeration also in Coronavirus disease 2019 (COVID-19) ARDS. This provides a strong rationale for its use in this scenario (15). Furthermore, Zarantonello and co-workers observed that PP induced an improvement of ventilation-perfusion matching and dorsal ventilation within the first 90 min (16). Another study established the positive role of PP on outcomes, in which shorter time to PP was associated with a decrease in mortality (17). Accordingly, COVID-19 guidelines also recommend PP in mechanically ventilated severe ARDS patients (18).

Despite the abundance of literature demonstrating the effects of PP immediately or after a short follow-up period in classical ARDS (12, 19, 20), its effect on lung volumes [i.e., end-expiratory lung volume (EELV)], respiratory mechanics and gas exchange over the recommended period, i.e., 16 h, was not previously elucidated in COVID-19 ARDS (21). Therefore, there is limited information on the optimal duration of PP in this population (22).

EELV is the Functional Residual Capacity measured in the presence of PEEP. It is dramatically reduced in ARDS (20, 23) because of loss of aeration which leads to severe hypoxemia (24). Quantitative EELV measurement can be performed by multiple breath nitrogen wash-in/wash out (NWI-WO) technique at the bedside without interrupting the mechanical ventilation. Additionally, it can accurately monitor PEEP or PP induced changes in the ARDS lung resting volume (25–27).

In this study we aimed at prospectively assessing how lung volumes, respiratory mechanics and gas exchange change before, during and after a 16-h cycle of PP in patients with COVID-19 ARDS. Furthermore, we explored whether changes in respiratory mechanics and gas exchange were correlated with changes in EELV during and after PP in this patient population.

## Materials and methods

This prospective cohort, physiological study was conducted in a single-center University Teaching Hospital in a 11-bed intensive care unit (ICU). The local ethical committee (Board Name: Ministry of Health University, Istanbul Research Hospital, Clinical Studies Ethical Committee. Reference no:18, Title: Evaluating the change in the end-expiratory lung volume after prone positioning in ARDS patients, date: 14.01.2021) reviewed and approved the study. Informed consent was waived based on the observational nature of the study. Study procedures were followed in accordance with the ethical standards on human experimentation according to the Helsinki Declaration of 1975. The study was registered at www.clinicaltrials.gov as NCT 04818164. The study was reported according to the recommendations of strengthening the reporting of observational studies in epidemiology (STROBE) (28).

### Patients

Inclusion criteria were as follows:

1. COVID-19 confirmed by severe acute respiratory syndrome coronavirus 2 (SARS-CoV-2) Polymerase Chain Reaction (PCR) molecular test;
2. Moderate-severe ARDS (ratio of arterial oxygen partial pressure to fractional inspired oxygen [PaO_2_/FiO_2_] < 200);
3. Intubation and mechanical ventilation.

All patients in the ICU that receive supplemental oxygen were reviewed daily by two investigators (i.e., OD, YD) whether they met the inclusion criteria.

Exclusion criteria were as follows:

1. Age < 18 years;
2. Invasive mechanical ventilation duration >12 h before study enrolment;
3. History of chronic obstructive pulmonary disease, or lung malignancies or resection;
4. Suspected or confirmed pulmonary embolism;
5. Pneumothorax or pneumomediastinum;
6. Chest drainage tubes;
7. Extra corporeal membrane oxygenation;
8. Hemodynamic instability (i.e., a cardiovascular Sequential Organ Failure Assessment [SOFA] score > 2);
9. Pregnancy.

As no previous studies evaluated the change on EELV in COVID ARDS, we assumed a rise in the PaO_2_/FiO_2_ for at least 16 mmHg after PP versus S1 to detect a two-sided significance of 0.05 and a power of 80% (29). With an effect size of 0.5 [(mean of group 1 - mean of group 2)/standard deviation of the control group], mean difference of 16 mmHg and a pooled standard deviation of 32, estimated sample size was calculated as 34 patients. Therefore, we decided to enrol a priori at least 40 patients in the study to account for possible missing data.

Enrolled patients spent 16 h in PP and were positioned back to SP as soon as they completed the cycle of PP. Measurements were performed at 7 time points: Supine (S) 1 (15 min after mechanical ventilation adjustments, immediately before turning to PP), Prone (P) 0 (15 min after turning to PP to allow stabilization), P4 (at 4 h of PP), P8 (at 8 h of PP), P12 (at 12 h of PP), P16 (at 16 h of PP) and finally S2 (15 min after turning to SP to allow stabilization; Supplementary Figure 1). Demographic data, laboratory data on the day of intubation (i.e., C-reactive protein, D-Dimer and ferritin), SOFA score, days since positive PCR confirmation of SARS-CoV-2, and variables about lung volumes, respiratory mechanics, ventilation, gas exchange and hemodynamics at all study timepoints were recorded. All patients included in the study were evaluated during their first prone position session.

### Study aims

Primary aim: to prospectively assess how lung volumes, respiratory mechanics and gas exchange change before, during and after a 16-h cycle of PP in patients with COVID-19 ARDS.

Secondary aims: to explore whether changes in respiratory mechanics and gas exchange were correlated with changes in EELV during and after PP in this patient population; to evaluate whether changes in lung volumes, ventilatory variables and gas exchanges could differ based on the response in respiratory mechanics (C_rs_) after PP in COVID-19 ARDS patients.

In order to assess changes in lung volumes, ventilatory variables and gas exchanges based on the change in the C_rs_ after PP we classified patients as responders versus non-responders to PP as follows:

1. As first, in each patient, arithmetic mean of C_rs_ during PP was calculated [i.e., (sum of 5 values obtained during PP)/5).] Subsequently, the mean of C_rs_ during PP was subtracted from the baseline C_rs_ value (S1). If the C_rs_ difference was greater than 0; patients were defined as responders. If the C_rs_ difference was lower than 0; then the patients were defined as non-responders.
2. Responders were patients with an average C_rs_ during 16 h of PP that was higher as compared with C_rs_ at baseline (i.e., supine position at the study start, S1);
3. Non-responders were patients with an average C_rs_ during 16 h of PP that was unchanged or lower as compared with C_rs_ at baseline (i.e., supine position at the study start, S1).

### Mechanical ventilation

Continuous infusion of endovenous anesthetic agents and rocuronium bromide was administered to set controlled mechanical ventilation. Pressure Control Ventilation with a ventilator capable to measure EELV via the NWI-WO technique (Carescape R860, General Electric, Madison, WI, USA) was used. The oxygenation goal was to keep a peripheral oxygen saturation (SpO_2_) 92% with a maximum of 80% FiO_2_. Detailed information about PEEP titration is reported in the Supplementary material. Briefly, at baseline, a range of PEEP values was applied for a pre-set duration in an incremental fashion (Supplementary Table 1). After each increment of PEEP, an automated EELV measurement was performed (30). PEEP with the highest EELV without decreasing the respiratory system compliance (C_rs_) was selected (i.e., OD, GYD) and applied throughout the entire study. If the PEEP level was changed for any reason after this time point, the patient was excluded from the analysis. No recruitment manoeuvres were performed between consequent PEEP levels and at the end of the PEEP setting procedure. The physician in charge of the patient was free to change the pressure control in order to match the starting tidal volume at S1.

### Study procedures and measurements

After changes in the body position (from S1 to PP, or from PP to S2), patients were maintained for 15 min in the new body position. This was confirmed by reaching a flat Carbon Dioxide Output (VCO_2_) trend. At baseline, central venous and arterial blood samples were drawn and blood gas analyses were then immediately performed. An expiratory hold manoeuvre was performed at each timepoint before EELV measurement in order to exclude the presence of dynamic hyperinflation. Afterward, ventilation and hemodynamic parameters were collected.

The following parameters were also collected from the mechanical ventilator at each timepoint. Tidal volume (V_t_), minute ventilation, inspiratory pressure above PEEP level (P_control_), mean airway pressure (P_mean_), Plateau airway pressure (P_plat_, measured after a two-second inspiratory hold), Static compliance of the respiratory system (C_rs_), FiO_2_, respiratory rate. Data acquired from the mechanical ventilator were averaged over 3 consecutive values and recorded real-time afterward. Tidal volume and minute ventilation were normalized by their respective predicted body weight (see below).

Lung Strain (31) was calculated as:


(1) $$\text{Strain}=\frac{V_{t}}{\text{EELV}}$$

where V_t_ is tidal volume and EELV is end-expiratory lung volume.

Alveolar ventilation and dead space were calculated according to the formula (32, 33):


(2) $$\dot{\text{V}}\,\text{E}=\dot{\text{V}}\,{\text{CO}}_{2}*\frac{1}{{\text{PaCO}}_{2}\,\left(1-\frac{V_{D}}{V_{T}}\right)}*863$$

$\dot{\text{V}}\,\text{E}$ is expired minute ventilation, $\dot{\text{V}}\,{\text{CO}}_{2}$ is carbon dioxide production per minute, PaCO_2_ is arterial carbon dioxide pressure (in mmHg) and $\frac{{\text{V}}_{\text{D}}}{{\text{V}}_{\text{T}}}$ is the ratio of dead space volume/tidal volume and 863 is the constant.

Ventilatory Ratio (VR) was computed from the equation:


(3) $$\text{VR}=\left(\mathrm{Minute}\,\mathrm{Ventilation}*{\text{PaCO}}_{2}\right)/\left(\mathrm{PBW}*37.5*100\right)$$

Predicted body weight (PBW) (kg) was calculated by using patient height measured when the patient was in flat position after intubation and with the following formula (34):


(4) $$\text{PBW}\,\left(\text{male}\right)=50+0.91\,\left({\text{height}}_{\text{cm}}-152.4\right)$$

$$\text{PBW}\,\left(\text{female}\right)=45.5+0.91\,\left({\text{height}}_{\text{cm}}-152.4\right)$$

### Statistical analysis

Data were expressed as median [Inter Quartile Range] and mean ± Standard Error of the mean as appropriate. Mixed-effect models with Benjamini–Krieger and Yekutielli correction for repeated measures were used to explore differences over time during PP and S2 as compared to S1. A non-linear regression curve of the percentage increase in the EELV/PBW, strain and PaO_2_/FiO_2_ was fitted to visualize the cumulative increase of each parameter as compared to S1. Mean values of the replicates were fitted with the least squares regression method and no weighting. No constraints were used to fit the curve. 95% Prediction Band of this curve was reported. Additionally, non-truncated violin plots of the percentage change in the EELV, strain and PaO_2_/FiO_2_ at P16 and S2 were superimposed with individual changes to reflect the data distribution. Pearson correlation coefficient was assessed to determine the relationship of a) strain and b) PaO_2_/FiO_2_ versus EELV/PBW. As for each patient multiple time-points were explored, robust clustering was performed by using each patient as cluster variable to take into account for within patient correlation. A linear regression analysis was performed to predict the change at P16 and at S2 as compared to S1 in strain and PaO_2_/FiO_2_ based on the change in EELV/PBW. Change in the lung volume, respiratory variables and gas exchanges stratified by responders versus non-responders in C_rs_ after PP were assessed using a 2-way ANOVA for repeated measurements. Difference in time, group and group be time interaction were reported. A two-tailed *p*-value < 0.05 was considered statistically significant. GraphPad Prism version 9.0 (Graphpad Software, San Diego, CA, USA) was used for the statistical analysis.

## Results

A total of 43 COVID-19 ARDS patients were enrolled in the study. Three patients were excluded from the analysis for the following reasons: 1 patient had a pneumothorax after the P12 time point and was treated with a chest tube. Extra Corporeal Membrane Oxygenation was initiated in another patient after P0. In 1 patient, EELV could not be accurately estimated despite repeated attempts (i.e., difference between measurements >20%). Forty patients were included in the final analysis. Patients were included at a median of 4 h [Inter Quartile Range, 2–5 h]. Patient characteristics are shown in Table 1.

**TABLE 1:** Patient characteristics at baseline.

Variables	Patients (*n* = 40)
**Baseline characteristics**	
Age	64.5 [58.3–74.8]
Sex (Male) *n*, %	22 (55)
Body mass index (kg/m^2^)	27.4 [24.7 – 30.4]
Predicted body weight (kg)	66.5 [52.4–74.7]
Comorbidities, *n* (%)	36 (90)
● Hypertension	● 30 (75)
● Diabetes	● 12 (30)
● Immune compromised	● 5 (13)
**Clinical illness severity**	
SOFA score (on the day of intubation)	6 [4–8]
Days since positive PCR SARS-CoV-2 confirmation	10 [7–16]
**Inflammatory biomarkers**	
CRP (mg/L)	111 [60–172]
D-Dimer (mg/L)	3 [1.4–10.8]
Ferritin (mg/L)	810 [328–1,734]

Data are presented as median and [Interquartile Range] or as count (proportion). SOFA, sequential organ failure assessment; CRP, C-reactive protein; PCR, polymerase chain reaction; SARS-CoV-2, severe acute respiratory distress syndrome coronavirus 2019.

### Effects of prone positioning on end-expiratory lung volume, strain and oxygenation

Absolute data of EELV, strain and oxygenation during the study period was reported in Table 2.

**TABLE 2:** Values of PaO_2_/FiO_2_, EELV/PBW and strain.

	S1	P0	P4	P8	P12	P16	S2
EELV (ml)	1,444 [1,065–1,759]	1,765 [1,399–2,015]*	1,980 [1,575–2,399]*	2,035 [1,685–2,408]*	2,082 [1,821–2,513]*	2,125 [1,838–2,535]*	1,582 [1,271–2,059]*
EELV/PBW (ml/kg)	23.4 [18.5–26.4]	25.3 [23.2–32.9]*	29.9 [26.1–35]*	30.7 [26.1–36.7]*	32.7 [28.3–38.4]*	33.5 [28.2–38.7]*	25.9 [20.1–31.6]*
Strain	0.31 [0.26–0.39]	0.25 [0.21–0.28]*	0.22 [0.19–0.27]*	0.22 [0.2–0.24]*	0.21 [0.18–0.25]*	0.20 [0.18–0.24]*	0.3 [0.24–0.35]
PaO_2_/FiO_2_	81 [65–97]	117 [87–167]*	130 [107–180]*	160 [118–192]*	163 [138–217]*	149 [122–228]*	106 [79–138]*

Data are presented as median and [Interquartile Range]. Mixed effect analysis with Benjamini–Krieger and Yekutielli correction was used for analysis. *p<0.05 versus S1. S1, Supine 1; P0, 0 h at Prone; P4, 4 h at Prone; P8, 8 h at Prone; P12, 12 h at Prone; P16, 16 h at Prone; S2, Supine 2; EELV/PBW, end-expiratory lung volume/Predicted body weight.

**FIGURE 1:**
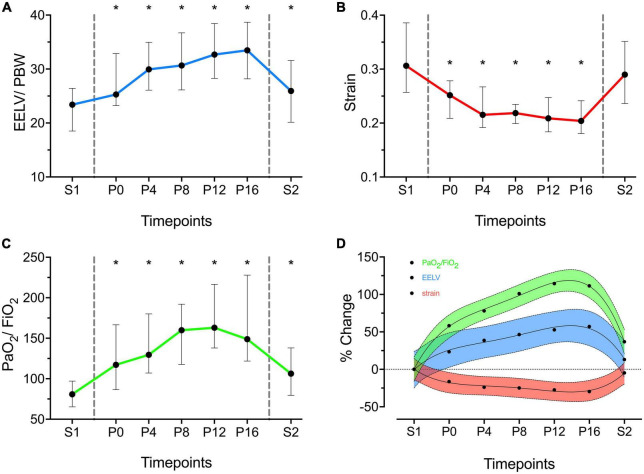
End-expiratory lung volume/predicted body weight (EELV/PBW – ml/kg) **(A)**, Strain **(B)** and PaO_2_/FiO_2_ **(C)** over time. X axis represents timepoints. Y axis represents absolute values of the variables. Data are presented as median and Inter Quartile Range. Areas within vertical dashed lines indicate prone position. *Statistically significant versus S1. **(D)** Cumulative percentage Increase in EELV/PBW (blue), Strain (red) and PaO_2_/FiO_2_ (green) as compared to S1 (S1 = 0%). Data are presented as mean and connecting regression line. Color shaded area indicates 95% Prediction Band of the regression line.

EELV/PBW and PaO_2_/FiO_2_ increased immediately after PP and decreased when PP was reversed (EELV/PBW P0 vs S1: 25.3 ml/kg [23.2–32.9] vs 23.4 ml/kg [18.5–26.4], *p* < 0.001; PaO_2_/FiO_2_ P0 vs S1: 117 [87–167] vs 81 [65–97], *p* = 0.002). However, both variables were higher at S2 as compared to S1 (EELV/PBW S2 vs S1: 25.9 ml/kg [20.1–31.6] vs 23.4 ml/kg [18.5–26.4], *p* = 0.006; PaO_2_/FiO_2_ S2 vs S1: 106 [79–138] vs 81 [65–97], *p* = 0.024). Strain declined rapidly after the onset of PP until P4 (P4 vs S1: 0.22 [0.19–0.27] vs 0.31 [0.26–0.39], *p* < 0.001). Subsequently, strain was stable until the end of PP (Table 2 and Figures 1A–C).

The absolute changes over time in EELV/PBW, strain and PaO_2_/FiO_2_ were reported in Figure 1. The cumulative and relative changes over time in EELV/PBW, strain and PaO_2_/FiO_2_ were reported in Supplementary Tables 2, 3, respectively.

The cumulative percentage change in the EELV/PBW was highest at P16 (+38.6% [22.2–76.6], *p* < 0.001; Supplementary Table 2). The highest relative EELV/PBW increase between consecutive steps was immediately after PP (i.e., between P0 and S1) when compared to other time, intervals (P0 vs S1: +15.8% [10.2–29.3], *p* < 0.001). Afterward change in the EELV/PBW declined progressively with a nadir between P12 and P16 (P16 vs P12: +3.7% [-0.2 to 9], *p* = 0.013; Supplementary Table 3).

Similarly, the highest cumulative percentage change in PaO_2_/FiO_2_ was at P12 (+92.8 [46.5–171.6], *p* < 0.001; Supplementary Table 2). Its greatest relative change was immediately after PP (P0 vs S1: +23.7% [14.1–49.8], *p* < 0.001). After P12, PaO_2_/FiO_2_ slightly decreased (P16 vs P12: -4.7% [-15.8–8.8], *p* = 0.5). (Supplementary Table 3).

### Association between end-expiratory lung volume versus strain and oxygenation

**FIGURE 2:**
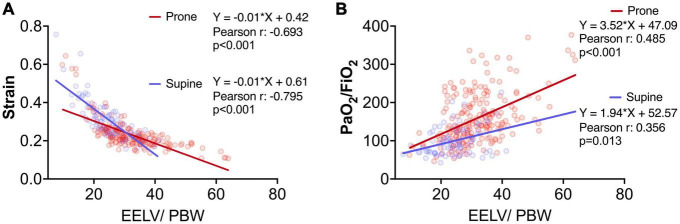
Association between end-expiratory lung volume/Predicted body weight (EELV/PBW – ml/kg) versus strain **(A)** and PaO_2_/FiO_2_ **(B)** (*n* = 280). Blue and red circles represent measurements during supine and prone position, respectively. Equations, Pearson correlation coefficient and *p*-value were reported. Measurements were adjusted by robust clustering for each study patient.

EELV/PBW was significantly correlated with strain and oxygenation, either in supine (2 timepoints) or in prone (5 timepoints) position (Figures 2A,B).

**FIGURE 3:**
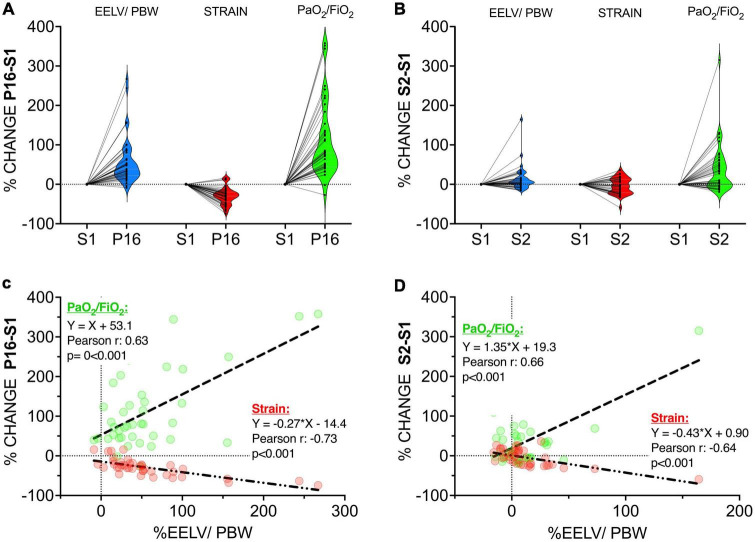
Upper Panel: Before-after plot of the percentage change from S1 (=%0) to P16 (end of prone position) **(A)** and from S1 (=%0) to S2 **(B)** (after turning back to supine position) in end-expiratory lung volume/Predicted Body Weight (EELV/PBW – ml/kg) (blue), strain (red), and PaO_2_/FiO_2_ (green). Dots represent individual values. Non-truncated violin plots are stacked to plot the distribution of each parameter. Lower Panel: Correlation coefficients and linear regression lines of the percentage change from S1 (=%0) to P16 (end of prone position) **(C)** and from S1 (=%0) to S2 (after turning back to supine position) **(D)** in EELV/PBW versus PaO_2_/FiO_2_ (green) and strain (red). Dashed lines represent PaO_2_/FiO_2_ and dash-dotted lines represent strain.

The changes in strain and oxygenation at the end of pronation (P16 versus S1; Figure 3A) and when patients were turned back to supine position (S2 versus S1; Figure 3B) were significantly associated with changes in EELV/PBW at the same timepoints (Figures 3C,D).

### Changes of respiratory mechanics, gas exchange and ventilatory parameters

C_rs_ decreased after PP (P0 vs S1: 29.5 ml/cmH_2_O [24.3–41.8] vs 32 ml/cmH_2_O [27.3–42.5], *p* < 0.001). C_rs_ increased only when the PP was reversed (S2 vs S1: 35 ml/cmH_2_O [28.3–45] vs 32 ml/cmH_2_O [27.3–42.5], *p* = 0.84). P_plateau_ was increased after PP and decreased when PP was reversed (P0 vs S1: 23.5 cmH_2_O [21–26] vs 22.5 cmH_2_O [21–25.8], *p* = 0.068; S2 vs S1: 23.5 cmH_2_O [21.3- 25] vs 22.5 cmH_2_O [21–25.8], *p* = 0.67). P_control_ was slightly increased during PP in order to maintain V_t_ stable. PaCO_2_, dead space percentage and ventilatory ratio were constant throughout the study period in the presence of a higher minute ventilation that was consequent to a higher set respiratory rate. (Table 3).

**TABLE 3:** Respiratory mechanics, gas exchange and ventilatory settings over time.

	S1	P0	P4	P8	P12	P16	S2
**Respiratory mechanics**							
*C*_rs_ (ml/cmH_2_O)	32 [27.3–42.5]	29.5 [24.3–41.8]*	31 [25–40.8]*	32 [25.3–40.8]*	31.5 [26–40.5]*	31.5 [26.5–39]*	35 [28.3–45]
*V*_t_ (ml)	437 [369–492]	411 [358–491]	431 [368–494]	454 [393–506]	463 [386–516]	461 [381–496]	462 [404–501]
*V*_*t*_/PBW (ml/kg)	6.8 [6.1–8]	6.4 [5.7–7.4]	6.9 [6.2–7.4]	7 [6.2–7.8]	7 [6.1–8.1]	7 [6.1–8.2]	7.1 [6–8]
**Ventilation**							
*P*_control_ (cmH_2_O)	16 [14.2–19]	16.5 [14.2–19]	16 [14–20]*	16 [14–21]*	16 [14–21]*	16 [14–20]*	15 [13.3–17.8]
*P*_mean_ (cmH_2_O)	16 [14–19]	17 [15–20]*	17 [15–20]*	17 [15–20]*	17 [15–20]*	17.5 [15–20]*	16 [14.3–20]
*P*_plateau_ (cmH_2_O)	23.5 [22–26.8]	24.5 [22–27]*	25 [23–28]*	25.5 [23–28.8]*	25.5 [23–28]*	25.5 [23–28]*	24.5 [22.3– 26]
Driving pressure	13 [12.3–15]	14 [13–16]*	14.5 [13–17]*	14 [13–17]*	14 [13–17]*	14 [13–17]*	13 [12–15]
RR (/min)	20 [17–24]	21 [18–24]*	22 [19–26]*	22 [20–27]*	22 [20–27]*	22 [20–26]*	20 [18–24]
MV (l/min)	8.9 [7.9–9.8]	8.9 [7.7–9.7]	9.5 [8.6–10.7]*	10.1 [9–11.8]*	10 [8.9–11.3]*	10.2 [8.6–11.3]*	9.1 [8.6–10.2]
MV/PBW (ml/min/kg)	141 [117–165]	137 [114–156]	156 [127–178]	165 [135–177]*	161 [133–183]*	151 [135–179]*	140 [124–177]
PEEP_extrinsic_ (cmH_2_O)	10 [8–12]	10 [8–12]	10 [8–12]	10 [8–12]	10 [8–12]	10 [8–12]	10 [8–12]
PEEP_intrinsic_ (cmH_2_O)	0 [0–0]	0 [0–0]	0 [0–0]	0 [0–0]	0 [0–0]	0 [0–0]	0 [0–0]
**Gas exchange**							
PaCO_2_ (mmHg)	49 [43–53]	50 [43–58]	48 [41–57]	47 [40–52]	45 [39–52]	46 [40–52]	46 [41–54]
Dead space (%)	67 [61–71]	68 [62–73]	68 [61–74]	67 [61–74]	66 [58–75]	67 [58–75]	66 [60–74]
Ventilatory ratio	1.8 [1.6–2.1]	1.8 [1.6–2.1]	1.8 [1.7–2.2]	1.9 [1.6–2.2]	1.8 [1.6–2.1]	1.8 [1.6–2.2]	1.7 [1.5–2.2]

Data are presented as median and [Interquartile Range]. Mixed effect analysis with Benjamini–Krieger and Yekutielli correction was used for analysis. *p<0.05 versus S1. S1, Supine 1; P0, 0 h at Prone; P4, 4 h at Prone; P8, 8 h at Prone; P12, 12 h at Prone; P16, 16 h at Prone; S2, Supine 2; EELV, end-expiratory lung volume; V_*t*_, tidal volume; PBW, predicted body weight; *C*_rs_, compliance of the respiratory system; *P*_control_, inspiratory pressure above PEEP level; *P*_mean_, mean airway pressure; *P*_plateau_, plateau airway pressure; RR, respiratory rate; MV, minute ventilation; PBW, predicted body weight.

The median PEEP level during the study was 10 [8–12] cmH_2_O.

Metabolic parameters are presented in the Supplementary Table 4.

### Lung volume, ventilatory variables and gas exchange stratified by responders versus non-responders in C_rs_ after prone position

Nine patients out of 40 (23%) were considered responders in terms of C_rs_ after a 16-h cycle of PP.

Mean C_rs_ of the overall patient population during S1 was 36.7 ml/cmH_2_0 and mean C_rs_ of the entire PP session was 33 ml/cmH_2_O.

**FIGURE 4:**
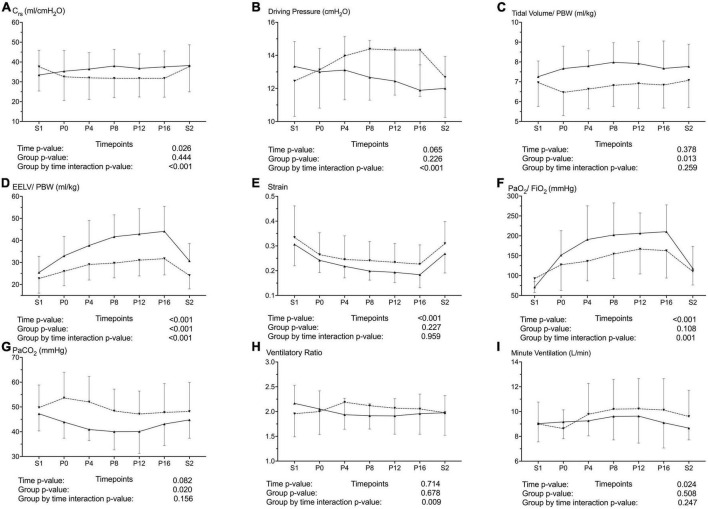
Changes in the resting lung volume, respiratory mechanics and gas exchange during the study time line and after stratification by the change in the respiratory system compliance (C_rs_) during prone position (PP) in COVID-19 ARDS patients. Upward triangles with solid lines represent increased C_rs_ during PP (responders, *n* = 9) and downward triangles with dashed lines represent Decreased or Equal C_rs_ during PP (non-responders, *n* = 31). *P*-values from the 2-way ANOVA for repeated measurements are provided below each variable. Data are presented as mean and unidirectional error bar. S1: supine position at baseline, 15 min after mechanical ventilation adjustments; P0, P4, P8, P12, P16: 15 min, 4, 8, 12 and 16 h of prone positioning; S2: 15 min after turning back to the supine position following a 16-h PP cycle. Upper Panel: Respiratory system compliance **(A)**, Driving Pressure **(B)**, tidal volume/Predicted body weight (PBW) **(C)**. Mid Panel: end-expiratory lung volume/Predicted body weight (EELV/PBW) **(D)**, Strain **(E)**, PaO_2_/FiO_2_ **(F)**. Lower Panel: Partial pressure of arterial carbon dioxide (PaCO_2_) **(G)**, Ventilatory Ratio **(H)**, minute ventilation **(I)**.

Responders had a lower driving pressure over time and a higher Vt during PP as compared with non-responders (Figures 4A–C). EELV/PBW was higher and increased more over time as compared with non-responders – and so was oxygenation; strain did not differ between the groups (Figures 4D–F).

Responders had a better CO_2_ clearance at the price of a similar minute ventilation. Consequently, ventilatory ratio decreased over time in responders as compared with non-responders (Figures 4G–I).

## Discussion

In this prospective cohort study in mechanically ventilated COVID-19 ARDS patients, we report the following main findings:

1. 16-h cycle of PP increases the end-expiratory lung volume, reduces the strain and increases the oxygenation. These changes occur early and rapidly after PP;
2. the changes in EELV/PBW, strain and oxygenation quickly decreased after returning to SP, although EELV/PBW and oxygenation remained different as compared to baseline SP;
3. both changes of strain and oxygenation at the end of PP and back to SP were significantly correlated with changes in EELV/PBW;
4. COVID-19 ARDS patients with an increase in C_rs_ after a 16h cycle of PP (i.e., responders) have a higher increase in EELV, a better oxygenation and CO_2_ clearance in the presence of a similar minute ventilation as compared with non-responders.

Prone position has an established survival benefit in moderate-severe ARDS. Several events, such as better distribution of ventilation-perfusion, increased gas-to-tissue ratio and increased chest wall elastance occur after switching from SP to PP (35). These transitions are crucial in reducing the stress and the strain delivered by the ventilator to the lung, which may contribute to mortality (36, 37). Cycles of ≥ 16 h of PP are recommended to minimize the exposure to potentially injurious ventilation during SP (12, 38). Previous studies evaluated the lung volume response immediately after PP or at the end of the PP duration in COVID-19 ARDS and the climax of the duration of lung volume change is unknown (15, 37, 39). We showed that EELV starts to increase rapidly after PP and peaks at the end of the 16-h cycle. Our findings support the use of PP cycle of at least 16 h as it is recommended in mechanically ventilated ARDS patients (40).

Increased lung strain and ventilation-perfusion uncoupling are directly associated with mortality (31, 41). As previously demonstrated, PP reduces non-aerated and over-aerated lung regions immediately and the inflated gas is more evenly distributed (31, 37). Furthermore, as recently reported by Zarantonello and co-workers, the ventilation-perfusion matching is improved early after PP (16). Consequently, the strain on the alveoli is reduced during the entire PP period as confirmed in our study. Therefore, PP should be used also to deliver protective mechanical ventilation.

Despite the sound evidence and rationale behind PP, it remains largely underutilized and reserved as a last resort against severe hypoxemia (42). Furthermore, the beneficial role of PP has been recently suggested in patients undergoing veno-venous ECMO (43) and in a time-dependent manner (44, 45). We observed a solid response to PP in terms of oxygenation. Although the time to peak in the PaO_2_/FiO_2_ was shorter as compared to the peak of the EELV, this response was preserved throughout the PP. The increase of oxygenation and decrease of strain at the end of PP or back to SP is consistent with the increase in the lung resting volume and this is clearly demonstrated by the robust correlation between the variables. Nevertheless, improved oxygenation after PP is weakly correlated with the decrease in the non-aerated lung regions (39, 41). In a recent study Protti et al. reported that despite an increase in oxygenation observed immediately after PP, the total gas volume of the lung was reduced in 73% of patients with COVID-19 ARDS (15). Additionally, better oxygenation response is not always associated with improved survival (4, 35). Therefore, the correlation between these two parameters may not necessarily indicate a causal relationship, at least immediately after PP.

Lung recruitment and derecruitment occur constantly during PP as a result of ongoing changes in the chest wall and lung elastance. Mainly, dorsal regions that contribute to hypoxemia are recruited while the ventral regions collapse due to the sponge like structure of the lungs and hydrostatic and gravitational forces (41, 46, 47). The net effect may differ significantly between patients and within a patient between consecutive PP sessions (12). Despite EELV improved after PP, C_rs_ was reduced in our study. Therefore, we cannot exclude that the mild decrease in the C_rs_ was due to alveolar overinflation despite median driving pressure was kept below 15 cmH_2_O through the study period (48, 49). However, the partitioned effects of PP on the chest wall and lung Compliance were not evaluated as an esophageal balloon was not available. Nonetheless, we can speculate that, although clinically negligible, decrease of the C_rs_ may be due to the worsening of the chest wall compliance. Indeed, decreased chest wall compliance during PP as compared to supine is well described (50). However, after stratifying patients based on the change in their C_rs_ during PP, we observed that approximately 1 out of 4 patients had an increase of C_rs_ over time during a 16h cycle of PP as compared with baseline (S1). Moreover, patients with improved C_rs_ had a higher EELV, a higher PaO_2_/FiO_2_ and a better CO_2_ clearance and compared with non-responders. Interestingly, in our study, for a similar change in minute ventilation, CO_2_ elimination more effective in responders. However, the definition of “C_rs_ responders versus non-responders” is arbitrary and we cannot exclude that some patients in the non-responder group may have had an increase in lung compliance despite a more significant decrease in the chest-wall compliance resulting in an overall decrease of C_rs_ (i.e., non-responders).

We acknowledge that our study has some limitations. We only focused our attention on the first PP session. Additionally, it may be argued that the time spent in PP was limited and extended durations may further contribute to lung recruitment. However, we believe that this phenomenon would be minimal as considered that after 12 h, the gain in EELV was negligible and the strain and oxygenation were stable. While it is possible that extended PP may further improve the EELV when the patient is back to SP after a prolonged PP session, its clinical relevance is questionable and its beneficial role is debated (51). Thirdly, the dead space estimation was burdened by the use of Heat and Moisture Exchange filters as active humidifiers were not available in our unit during the COVID pandemic. Fourth, partitioned respiratory mechanics could not be assessed as the esophageal balloon was not available. As last, advanced experimental techniques of lung imaging such as electrical impedance tomography (52) were not available, therefore the redistribution of the tidal ventilation due to the increase in the resting lung volume during PP versus SP could not be assessed.

- In conclusion, early PP rapidly increases the end-expiratory lung volume, decreases the lung strain and leads to a higher oxygenation in COVID-19 ARDS. Changes in the strain and PaO_2_/FiO_2_ are robustly correlated with the change in the EELV. A higher respiratory system compliance during PP may suggest an increased EELV and better gas exchange. Future studies should evaluate whether the change in lung volume may be maintained over a longer period of time and whether the increase in EELV during PP may predict a favorable outcome in patients with COVID-19 ARDS.

## Data availability statement

The original contributions presented in this study are included in the article/Supplementary material, further inquiries can be directed to the corresponding author.

## Ethics statement

The studies involving human participants were reviewed and approved by Ministry of Health University, Istanbul Research Hospital, Clinical Studies Ethical Committee. Reference no: 18, Title: Evaluating the change in the end-expiratory lung volume after prone positioning in ARDS patients, date: 14.01.2021. The ethics committee waived the requirement of written informed consent for participation.

## Author contributions

ODi designed the work and drafted the first manuscript. ER revised the first draft, analysed, and interpreted the data. ODi, ODe, GY, SÜ, and YD were the major contributor to acquire and interpret the data. All authors contributed to the writing of the manuscript, read, and approved the final version of the manuscript.

## Abbreviations

ARDS: acute respiratory distress syndrome
COVID-19: Coronavirus disease 2019
C_*rs*_: respiratory system compliance
EELV: end-expiratory lung volume
MV: minute ventilation
NWI-WO: nitrogen wash-in/wash-out
PaCO_2_: arterial carbon dioxide pressure
PaO_2_/FiO_2_: ratio of arterial oxygenation partial pressure to fractional inspired oxygen
PBW: predicted body weight
PCR: polymerase chain reaction
PEEP: positive end-expiratory pressure
PP: prone position
SARS-CoV-2: severe acute respiratory distress syndrome coronavirus 2
SOFA: sequential organ failure assessment
SP: supine position
VCO2: carbon dioxide output
$\dot{\text{V}}\,C\,O_{2}$: carbon dioxide output per minute
VD/VT: ratio of dead space volume/tidal volume
$\dot{\text{V}}\,E$: expired minute ventilation volume
VR: ventilatory ratio
V_t_: tidal volume.
