# Supplementary material for: Effect of prone positioning on end-expiratory lung volume, strain and oxygenation change over time in COVID-19 acute respiratory distress syndrome: A prospective physiological study
Source: Front Med (Lausanne). 2022 Dec 2;9:1056766. doi: 10.3389/fmed.2022.1056766 (PMC9755177; doi:10.3389/fmed.2022.1056766)
Supplement: Supplementary file 1 [file Data_Sheet_1.pdf]

PEEP titration procedure:

Oxygenation goal was to keep $SpO_2 \geq 92\%$ with the PEEP set at 5 cm H_2O and the Fraction of the inspired oxygen (FiO_2) is set at 80% during Supine Position (SP). PEEP titration was performed with the automated module incorporated to the mechanical ventilator (PEEP InView, Carescape R860, General Electronics, Madison, WI, USA) in order to achieve the target oxygenation that does not compromise the respiratory system compliance (Crs). This function increases PEEP from a desired level and performs a Crs measurement with a two second inspiratory hold maneuver and an end-expiratory lung volume (EELV) measurement after each PEEP step. Initial and final PEEP levels can be set. Additionally, duration of each step can be defined. If there was a disagreement greater than 20% between the EELV measurements within a time point, the measurement was discarded (as per the manufacturer's instructions) and a new EELV measurement was performed. If a stable measurement cannot be performed despite 3 attempts, the patient was excluded from the study.

We initiated the PEEP trials with the following settings:

Initial PEEP: 6

Final PEEP: 18.

5 steps. Each step 5 minutes.

To avoid hypoxemia, FiO_2 was increased by 0.1 to measure lung volumes (i.e., from 0.8 to 0.9).

The PEEP level that achieved a $SpO_2 \geq 92\%$ with best Crs and highest EELV was set. We did not apply higher PEEP levels higher than 18 cm H_2O as our previous clinical experience showed that precise EELV measurements cannot be achieved due to air leaks.

PEEP levels set for the patients in the study were:

Supplemental Table 1: PEEP levels set at baseline.

PEEP cm H_2O	*Number of patients*
6	1
7	0
8	17
9	0
10	6
11	1
12	6
13	1
14	4
15	2
16	1
17	0
18	1

After setting the PEEP level, other mechanical ventilation parameters were set as follows:

Inspiratory pressure was set to the value that resulted in a tidal volume of <8 ml/ Predicted Body Weight with P_{plat} < 30 cmH_2O. I/E was set according to the time constants. A minimum of 3 times the value of the time constant was set for the time cycling. All of these parameters (except the PEEP level and the mode of the ventilation) were adjusted as required, every 4 hours and after every position change. No recruitment maneuvers were performed during the study period.

A closed suction catheter, a Heat and Moisture Exchange filter (Twinstar 55, Draeger, Germany), and a side-stream capnograph was attached from distal to the proximal of the Y-piece. Instrumental dead space incurred by these additions were: ~4 ml, 9,5 ml and 55 ml,

respectively. Patients were ventilated in 5 degrees inclined position, both during SP and PP. No suction was allowed 30 minutes prior to EELV measurement. If the trachea was suctioned or there was an inadvertent disconnection from the ventilator during this time period, patient was ventilated for 30 minutes before the measurement was performed.

Supplemental Figure 1: Study timeline.

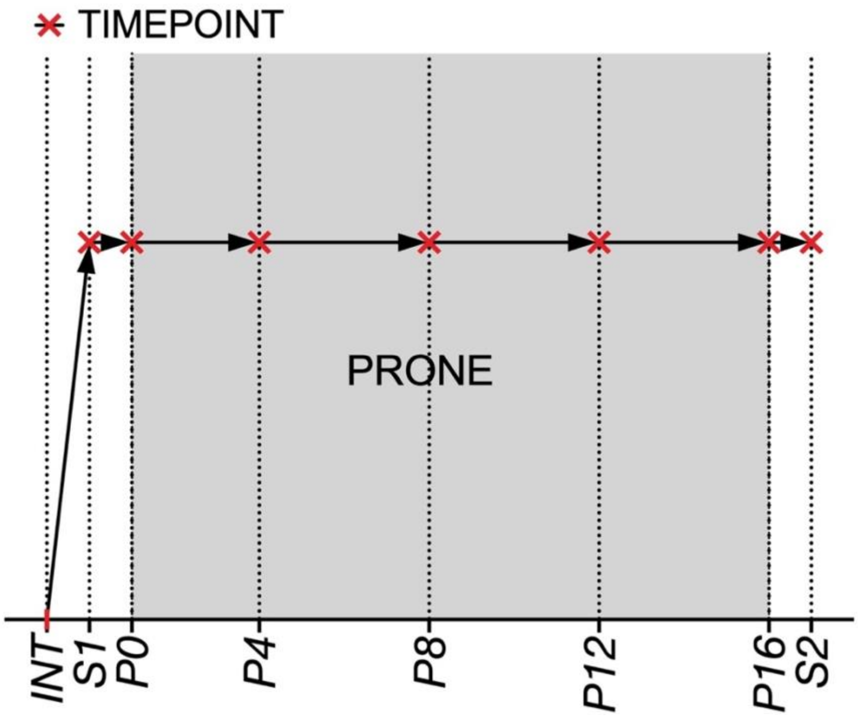

Int: Endotracheal Intubation.

Supplemental Table 2: Cumulative percentage change compared to Supine 1 in PaO_2/FiO_2, End-Expiratory Lung Volume (EELV) / Predicted Body Weight (PBW) and strain over time.

	P0	P4	P8	P12	P16	S2
**%PaO_2/FiO_2**	23.7 [8.1 to 102]	50.7 [24.7 to 113.4]	82.8 [36.8 to 136.1]	92.8 [46.5 to 171.6]	79.2 [44 to 149.3]	24.3 [-5.5 to 58.9]
**% EELV/ PBW**	15.8 [-0.2 to 40.2]	31.6 [10.4 to 48.6]	35.4 [15.2 to 64.9]	37 [19.5 to 72.6]	38.6 [22.2 to 76.6]	5.8 [-2 to 16.7]

% Strain	-19.8 [-34.4 to -4.7]	-25.7 [-39.3 to -12.9]	-25.2 [-39.3 to -9.8]	-27.1 [-42.9 to -14.3]	-30 [-44.7 to -19.7]	-4.9 [-22 to 12.3]

Supplemental Table 3: Comparison of the median difference (relative change) and [95% Confidence Interval of the median difference] of the percentage change compared to Supine 1 in PaO_2/FiO_2, End-Expiratory Lung Volume (EELV) / Predicted Body Weight (PBW) and strain over time.

	P0 vs S1	P4 vs P0	P8 vs P4	P12 vs P8	P16 vs P12	S2 vs P16
**% PaO_2/FiO_2**	23.7 [14.1 to 49.8]	18.4 [5.7 to 30.4]	14.6 [2.5 to 31.2]	9.7 [-8.2 to 27.4]	-4.7 [-15.8 to 8.8]	-60 [-94.3 to -37]
**% EELV/PBW**	15.8 [10.2 to 29.3]	15.1 [9.4 to 21.1]	7.2 [2.9 to 9.2]	4.1 [1.8 to 7.7]	3.7 [-0.2 to 9]	-35.4 [-52.5 to -24]
**% Strain**	-19.8 [-28.2 to -13.3]	-6.3 [-12 to -0.1]	-1.9 [-4.5 to 1.3]	-2.5 [-6.3 to 0.7]	-1.5 [-3.7 to 0.9]	26.5 [20.5 to 30.6]

Supplemental Table 4: Metabolic Parameters over time. Data are presented as median [Interquartile Range]. Oxygen saturation of the central venous blood. $P_{v-a}CO_2$: Carbon dioxide gap.

	S1	P0	P4	P8	P12	P16	S2

**Lactate (mmol/L)**	1.8 [1.23-2.5]	1.7 [1.3-2.3]	1.7 [1.3-2.2]	1.7 [1.3-2.1]	1.7 [1.4-2.4]	1.7 [1.4-2.2]	1.6 [1.4-2.7]
**pH**	7.35 [7.27-7.41]	7.34 [7.26-7.41]	7.33 [7.28-7.41]	7.35 [7.29-7.45]	7.35 [7.33-7.43]	7.36 [7.31-7.43]	7.37 [7.30-7.40]
**$S_{cv}O_2$ (%)**	71 [64-79]	76 [69-83]	79 [73-86]	80 [74-84]	80 [71-84]	78 [69-82]	74 [65-81]
**$P_{v-a}CO_2$ (mmHg)**	5.6 [3.9-6.9]	6.1 [4.5-8]	6.1 [4.9-8.3]	6.5 [4.7-7.9]	6.5 [5.3-9.7]	6.8 [5.3-9]	6.4 [4.8-7.6]

Supplemental Table 5: Fraction of inspired oxygen (FiO_2) over time. Data are presented as median [Interquartile Range]. Repeated Measures Friedman Test with Benjamini-Yekutielli-Krieger correction for multiple comparison. *: p<0.05 vs Supine1

	S1	P0	P4	P8	P12	P16	S2
FiO_2	0.8 [0.8-0.8]	0.8* [0.6-0.8]	0.6* [0.5-0.8]	0.6* [0.5-0.775]	0.6* [0.4-0.675]	0.5* [0.4-0.6]	0.6* [0.5-0.8]
